# Wearable-derived heart rate variability responses to a mind-body exercise intervention in older people from high southern latitudes (Chilean Patagonia, ~53°S)

**DOI:** 10.3389/fphys.2026.1794752

**Published:** 2026-08-28

**Authors:** Diego Mabe-Castro, Ruby Méndez-Muñoz, Pablo Sanhueza, Matías Castillo-Aguilar, Ginés Viscor, Antonio Zamuner, Cristian Núñez-Espinosa

**Affiliations:** 1 Centro Asistencial Docente e Investigación, Universidad de Magallanes (UMAG), Punta Arenas, Chile; 2 Escuela de Medicina, Universidad de Magallanes (UMAG), Punta Arenas, Chile; 3 Secció de Fisiologia, Departament de Biologia Cel·lular, Fisiologia i Immunologia, Facultat de Biologia, Universitat de Barcelona, Barcelona, Spain; 4 Laboratorio de Investigación Clínica en Kinesiología, Departamento de Kinesiología, Universidad Católica del Maule, Talca, Chile; 5 Centro de Investigación en Neuropsicología y Neurociencias (CINPSI Neurocog), Universidad Católica del Maule, Talca, Chile

**Keywords:** aged, exercise therapy, heart rate variability, mind-body, wearable electronic device

## Abstract

**Background:**

High southern latitudes expose older adults to chronic cold and marked seasonal light variability, potentially challenging physiological resilience. Yet, few interventions have examined stress-related autonomic regulation in these geographically extreme contexts, and wearable-derived heart rate variability (HRV) frameworks remain insufficiently tested.

**Objective:**

To evaluate a wearable-derived HRV framework, including signal quality control and Bayesian multivariate modeling, to characterize resting and exercise-evoked autonomic responses before and after a low-dose mind–body exercise (MBE) program in older adults living at high southern latitude.

**Methods:**

Community-dwelling older adults completed a 24-week MBE intervention. RR intervals were continuously recorded using a chest-worn wearable device. HRV features were extracted from quality-controlled 5-minute segments at rest and after a standardized functional exercise bout. Intervention effects and potential moderators, including age, sex, body composition, and educational level, were estimated using multivariate Bayesian models to characterize individual variability and generate probabilistic estimates in a small sample.

**Results:**

The intervention did not produce uniform resting HRV changes (RMSSD: β = −0.14, 95% CI [−1.41, 1.06]). Instead, modeling revealed a reorganization of exercise-evoked autonomic responses, including changes in sympathetic modulation (SNS index: β = −1.63, 95% CI [−3.05, −0.24]), alongside marked inter-individual heterogeneity. Age, sex, and adiposity-related measures consistently moderated autonomic response patterns.

**Conclusion:**

In older adults living at high southern latitudes, a low-dose MBE program was associated with changes in cardiac autonomic regulation, particularly in acute exercise-evoked reactivity rather than resting HRV. This framework supports future longitudinal real-life monitoring integrating environmental covariates to improve individualized risk and response profiling.

## Introduction

Wearable technologies allow the assessment of physiological regulation using portable, non-invasive sensors, expanding the possibilities for studying human responses to environmental stressors beyond highly controlled instrumentation (Furlan et al., 2000; Mao et al., 2023; Srivastava and Yadav, 2025). Heart Rate Variability (HRV) is a widely used marker of cardiac autonomic regulation and is particularly well-suited for wearable-based acquisition under standardized experimental conditions (Electrophysiology TF of the ES of C the NA, 1996; Li et al., 2023; Quigley et al., 2024). While research on extreme environments has largely been focused on heat or altitude, environmental extremity is not limited to these conditions. Populations living at high southern latitudes are chronically exposed to distinct environmental challenges, including cold temperatures, inter-seasonal pronounced seasonal light variability, and even isolation (Mabe-Castro et al., 2024b; Castillo-Aguilar et al., 2025a; Klos et al., 2025). Punta Arenas or Ushuaia (the most southerly inhabited cities in the world) lie much less poleward than Iceland or northern Norway in the Northern hemisphere. However, southern Chilean and Argentine Patagonia still qualifies as an extreme environment because of its cold oceanic–subpolar climate, with annual means around 5–7 °C, which are substantially lower than those of many cities at comparable latitudes in the Northern hemisphere. Persistent strong winds greatly enhance wind chill and make outdoor activities and logistics challenging. Together with cold seas (Humboldt and Antarctic Circumpolar Currents), high humidity, rapidly changing weather, and geographic isolation, this produces harsh living and working conditions despite their moderate latitude. During aging, the ability of the autonomic nervous system to adapt to internal or external challenges declines, causing reduced HRV and a blunted cardiovascular adaptability to these environmental conditions; however, few interventions have been evaluated to address the physiological alterations associated with chronic exposure to such environmentally challenging contexts (Olivieri et al., 2024; Cosarderelioglu et al., 2025).

Exercise-based interventions are among the most consistently studied strategies to support autonomic regulation in older adults, with evidence suggesting both acute and training-related effects on HRV (Grässler et al., 2021; Yang et al., 2024; Zhang et al., 2025). In particular, acute bouts of exercise typically elicit transient reductions in vagally mediated HRV indices, followed by recovery dynamics that reflect autonomic flexibility and adaptation, characteristics that tend to decrease with aging (Olivieri et al., 2024). Mind-Body Exercise (MBE) modalities integrate physical movement with attentional control and breathing regulation, mechanisms that are theoretically relevant for autonomic regulation (Mancia et al., 2007; Shi et al., 2022; Dong et al., 2024). Yet, existing evidence remains heterogeneous, with reported effects varying across HRV domains, intervention dosification, time scales, and individual characteristics (Zou et al., 2018). Importantly, most intervention studies have been conducted in non-chronically exposed individuals to extreme environments and have rarely examined how acute autonomic responses and regulations are modified after a period of MBE intervention, nor how participant-level factors shape these responses. Nevertheless, evidence from randomized trials synthesized in a systematic review and meta-analysis suggests that longer-term Tai Chi/Yoga interventions can improve HRV indices (normalized LF/HF metrics and LF/HF ratio), alongside reductions in perceived stress, in non-extreme settings (Bernardi et al., 2001; Zou et al., 2018). As a result, the extent to which MBE interventions can modulate both overall and exercise-induced autonomic responses in extreme environments remains insufficiently understood.

Accordingly, the present study examined autonomic regulation at two complementary levels: (i) chronic (training-related) change, defined as the pre-to-post 24-week shift in HRV, (ii) acute reactivity, defined as the within-visit change from resting HRV to post–functional-test recovery HRV and (iii) the interaction effect between the acute response prior to the intervention and the response following the intervention. Our primary aim was to test whether the intervention reorganized acute autonomic responsiveness (Phase × Condition interaction), rather than producing uniform resting HRV shifts. We further explored the extent to which age, sex, and body composition shaped both chronic effects and acute responses, using wearable-derived HRV measures.

## Materials and methods

### Study design

We conducted a feasibility-oriented, pre-experimental study using a single-arm pre–post design. A total of 44 community-dwelling older adults were recruited at the CADI-UMAG Teaching and Research Center (Centro Asistencial Docente e Investigación) and evaluated at baseline and again after a 24-week intervention. All participants received the same program (no parallel control group), and the analytical focus was the within-participant change between time points.

Sample size was set pragmatically for a feasibility stage while ensuring interpretable within-person effects under a paired design.

### Participants

Older adults (≥60 years) permanently living in Punta Arenas, Chile, were recruited via non-probabilistic convenience sampling within a physical–cognitive stimulation program. Written informed consent was obtained. Inclusion required independent ambulation (assistive devices allowed), ability to follow multi-step instructions, no diagnosis of dementia/major neurocognitive disorder, and no severe cognitive impairment on screening, stable medication for ≥4 weeks, and availability for weekly sessions over 24 weeks. Exclusion criteria included contraindications to moderate exercise; unstable cardiometabolic/pulmonary disease (myocardial infarction or stroke <6 months, decompensated heart failure, uncontrolled arrhythmias, severe valvular disease, unstable angina, uncontrolled BP ≥140/100 mmHg); and the use of medications with known major effects on autonomic nervous system activity β-blocker therapy was permitted only if clinically indicated and stable for ≥4 weeks; recent initiation or dose changes were exclusionary; musculoskeletal conditions limiting safe participation; current participation in a similar supervised mind–body program; severe unstable psychiatric disorder or recent substance abuse disorder; uncorrected sensory deficits preventing instruction-following; and planned relocation/prolonged absence. All participants underwent a standardized anamnesis to collect demographic and clinical information, including age and sex, comorbidities, educational level (schooling), and medication use; these participant-level characteristics were subsequently included as covariates in the analyses. Attendance logs were used to quantify adherence; per-protocol was ≥80% attendance. The intention-to-treat analysis included all participants with at least one post-baseline assessment to preserve within-person information under the paired design, while missing observations were accounted for within the Bayesian modeling framework.

### Mind–body exercise intervention

Participants completed a standardized, instructor-led MBE program delivered indoors in small groups (8–12 participants), consisting of 24 supervised sessions (one 60-minute session per week) over 24 weeks. The post-intervention data collection was conducted uninterrupted within two weeks of the last session.

The intervention was designed as a low-dose option for high-latitude settings, integrating paced breathing, mindful attention, and slow coordinated movements to progressively train mobility, balance, functional strength, and postural control, prioritizing movement quality over heart-rate targeting in a comfortable-temperature setting (21-22 °C). Each session followed a fixed structure: seated diaphragmatic breathing-centering (5 min), gentle joint mobility and dynamic warm-up (10 min), multi-planar balance and gait sequences mainly performed standing (20 min) with occasional dual-task coordination, chair-based strengthening emphasizing lower-limb and trunk function using body weight and elastic bands (15 min), and a mindfulness-oriented cool-down including breathing, body scan, and brief guided imagery (10 min). Intensity was monitored using self-perceived physical exertion (from 0, no physical exertion, to 10, maximal physical exertion) maintained within a light-to-moderate effort range (transformed target Borg RPE 11–13) (Borg, 1998).

To support intervention fidelity, instructors used a standardized manual and session checklist specifying sequence, duration, and key qualitative cues for each block; adherence was monitored via attendance logs (> 80% considered adequate per protocol definition). The program was structured across three progressive phases (weeks 1–8 adaptation, 9–16 consolidation, 17–24 progressive), with incremental adjustments in breathing cycle length/attentional demands, multi-directional mobility, reduced base of support, and higher dual-task load in gait-balance exercises, and increased repetitions and band resistance in strengthening (i.e., higher-level elastic bands).

### Heart rate variability

All participants were evaluated between 09:00 and 11:00 a.m. to minimize potential circadian influences. Prior to the assessments, participants were familiarized with the experimental protocol and instructed to abstain from stimulants (e.g., coffee, tea, and soft drinks), alcoholic beverages, and strenuous physical activity for 24 hours before testing. They were also advised to consume a light meal at least 2 hours before the evaluation and to empty their bladder prior to testing.

**Figure 1:**
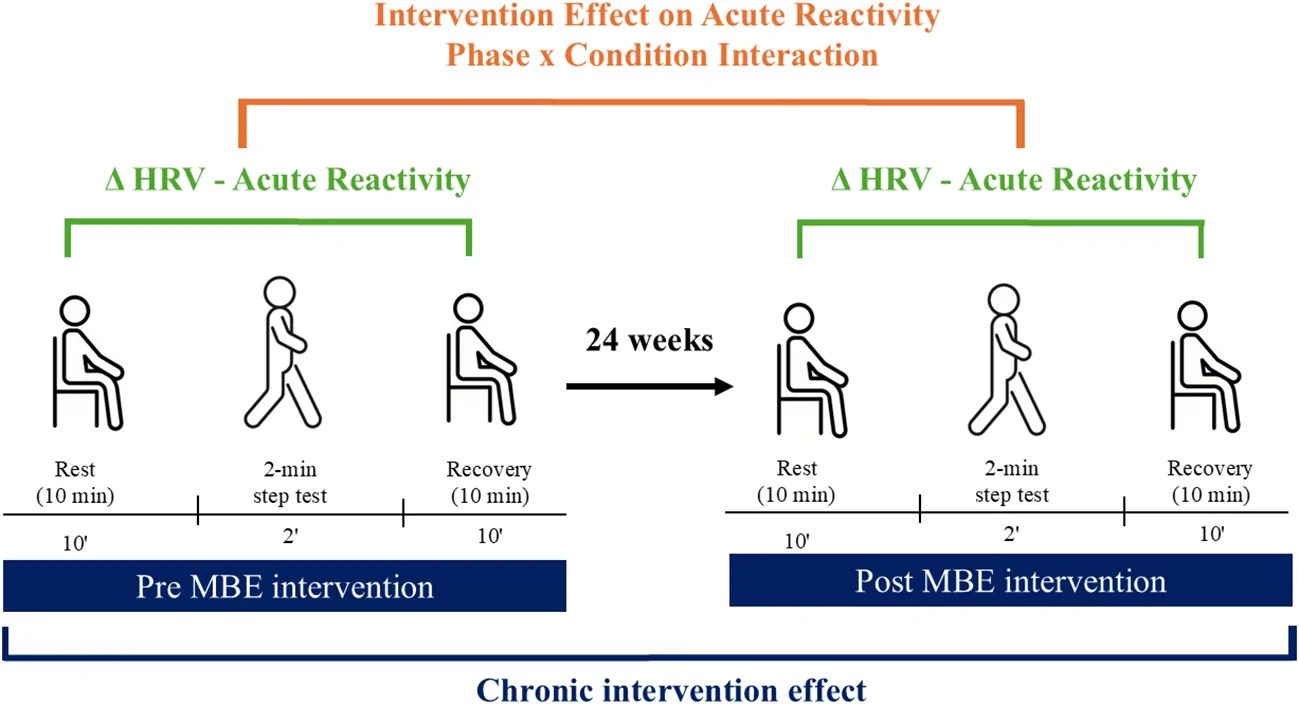
Methodological diagram explaining the acute and chronic effects evaluated from HRV data.

HRV was derived from R–R interval recordings collected with the Polar Team2 system (Polar^®^, Finland). Continuous beat-to-beat recordings were obtained during a 10-minute period while participants rested in a sitting position. A 5-minute artifact-free segment was selected for analysis (Castillo-Aguilar et al., 2021). HRV was assessed at two time points, before (baseline) and after the 24-week intervention, using the same standardized recording procedure at both visits. At each visit, participants first completed a resting RR recording, from which a 5-min artifact-free segment was selected. Participants then performed a brief functional test: the two-minute step test subtest; Senior Fitness Test format (TMST). Immediately after, participants returned to a seated position and underwent an additional 10-minute interval recording. To minimize the influence of the immediate acute cardiovascular response to exercise and allow partial stabilization of cardiac autonomic activity, the post-exercise HRV analysis was based on a 5-min artifact-free segment of the recovery period (Zou et al., 2018) (see **Figure 1**). Signal processing and HRV computation were performed in Kubios HRV^®^ (Kuopio, Finland). Automated artifact correction was applied using the Low correction setting with cubic-spline interpolation. Segments were excluded if > 5% of beats required correction, while segments with ≤5% corrected beats were retained, and the corrected-beat percentage was documented. Detrending used the Kubios default smoothness-priors polynomial approach (order 3; default λ settings for 5 min data). Time-domain indices included the Root Mean Square of Successive RR interval Differences (RMSSD, ms) and the standard deviation of RR intervals (SDNN, ms) (Bernston et al., 1997; Buchheit and Gindre, 2006; Buchheit et al., 2010). In addition, Kubios-derived composite indices were extracted (PNS and SNS indices) alongside Baevsky’s Stress Index, following the software’s implementation (PNS based on mean R–R, RMSSD, and Poincaré Plot Index SD1 in Normalized Units; SNS based on mean R–R, Stress Index, and Poincaré Plot Index SD2 in Normalized Units). Frequency-domain estimates were computed using a Welch periodogram (Hanning window, 50% overlap) after resampling at 4 Hz, integrating standard spectral bands: VLF 0.0033–0.04 Hz, LF 0.04–0.15 Hz, and HF 0.15–0.40 Hz. Absolute power (ms²) was reported and log-transformed when appropriate. Respiration was spontaneous; therefore, respiratory rate and the spectral peak within the HF band were inspected to ensure that the dominant respiratory-related oscillation remained within the conventional HF range (0.15–0.40 Hz). This approach is commonly used in short-term HRV studies when paced breathing is not implemented. Artifacts and ectopic heartbeats (which did not exceed 3% of the recorded data) were excluded (Electrophysiology TF of the ES of C the NA, 1996; Quigley et al., 2024).

### Body composition assessment

Body composition was assessed by bioelectrical impedance analysis (BIA) using a Tanita body composition analyzer (Ironman InnerScan BC-588, Tanita Corp., Tokyo, Japan). Participants were measured barefoot and with light clothing, after previously voiding the bladder, and were asked to avoid vigorous exercise and alcohol for 24 h and to refrain from large meals and caffeine for at least 3–4 h before assessment; between 09:00 and 11:00 h to reduce hydration-related variability. Height was measured with a wall-mounted stadiometer (to the nearest 0.1 cm), and body mass was obtained from the Tanita scale (to the nearest 0.1 kg). From these data, BMI (kg/m²) was calculated. The primary BIA-derived outputs were fat mass (kg), body fat (kg), and fat-free mass (kg); additional Tanita indices (e.g., skeletal muscle mass, visceral fat rating) were extracted if available from the device model and reported consistently across participants.

### Statistical analysis

A Bayesian framework was employed to characterize changes in our variables of interest following the MBE intervention. Bayesian inference was preferred over traditional frequentist methods as it provides complete posterior distributions for all parameters, thereby allowing for a full quantification of uncertainty and a probabilistic interpretation via credible intervals, instead of rigid p-value limits for significance interpretation (van de Schoot et al., 2021).

Descriptive statistics were reported as mean and standard deviation (M ± SD) for continuous variables, after assessing the distribution characteristics through graphical inspection. For categorical variables, absolute and relative frequencies (n [%]) were used.

To measure pre-to-post intervention changes in the HRV parameters in response to exercise, we fitted three Multivariate Bayesian linear mixed-effects models, one for each of HRV analysis domain (time, frequency and composite measures of HRV), while accounting for residual correlation between response variables. In these models, exercise (pre/post exercise) and the interaction with the both acute intervention (pre/post intervention) were specified as the main fixed effects, adjusting for age, sex, body composition and BMI as covariates. Interactions between main effects and these covariates were also included to assess their influence on the intervention’s effect. Subject ID was included as a random effect to capture and isolate inter-individual variance from the main fixed effects.

Weakly informative priors, centered on a null effect, were used to exert a regularizing effect, reducing the influence of outliers and stochastic noise. All models were fitted using the No-U-Turn Sampler (NUTS) algorithm, a variant of Hamiltonian Monte Carlo (HMC). We ran 4 chains, with 10,000 warm-up iterations and 10,000 effective iterations per chain. Model convergence was confirmed by ensuring an R-hat statistic < 1.01 and an effective sample size (ESS) > 2,000, and by visually inspecting trace plots to confirm convergence to a stationary distribution.

Inference followed the SEXIT (Sequential Effect eXistence and Significance Testing) framework. For each estimated parameter and its corresponding posterior distribution, we report the posterior median and its 95% highest-density credible interval (HDI). We also present the probability of direction (pd) as an index of effect existence, representing the probability that an effect goes in a particular direction (positive or negative) and ranging from 50% to 100%. Practical significance (ps) was reported as the probability that the effect is outside the region of practical equivalence (ROPE). The ROPE was defined as ±0.1 times the standard deviation of the response variable, and to ensure consistency all predictors were standardized before modeling.

## Results

### Sample characterization

A total of 44 community-dwelling older adults were included (29 women, 15 men; mean age 69.7 ± 5.30 years) All the participants were included in the final analyses (100% retention). Age did not differ between sexes. In contrast, men presented higher body weight and height compared with women. BMI was marginally elevated in both groups and showed no clear sex difference. The rest of the sample characteristics at baseline can be observed in **Table 1**.

**Table 1:** Sociodemographic characteristic of the recruited participants at baseline.

Characteristic	Female N = 29	Male N = 15	Difference	95% CI
Age (years old)	69.90 ± 5.22	69.33 ± 5.63	0.11	-0.52, 0.73
Comorbidities (n)				
Diabetes	10 (34%)	7 (47%)		
Hypertension	15 (52%)	9 (60%)		
Dislipidemia	8 (28%)	4 (27%)		
Asthma	3 (10%)	2 (13%)		
Weight (kg)	73.89 ± 14.51	86.59 ± 17.93	-0.80	-1.5, -0.14
Height (cm)	156.07 ± 7.88	165.50 ± 5.93	-1.4	-2.1, -0.64
BMI (kg/m²)	30.16 ± 5.73	31.48 ± 5.66	-0.24	-0.92, 0.44
Body fat (kg)	29.58 ± 10.30	29.77 ± 12.71	-0.02	-0.65, 0.62
Muscle mass (kg)	41.97 ± 5.82	56.57 ± 7.67	-2.2	-3.0, -1.4
Body water (%)	44.40 ± 4.81	49.95 ± 5.55	-1.1	-1.8, -0.43
Bone mass (kg)	2.25 ± 0.31	2.97 ± 0.37	-2.2	-3.0, -1.4
HF power (ms²)	137.93 ± 211.00	80.27 ± 119.05	0.34	-0.28, 0.97
LF power (ms²)	306.52 ± 367.20	210.60 ± 217.25	0.32	-0.30, 0.95
VLF power (ms²)	59.31 ± 64.49	64.07 ± 109.20	-0.05	-0.68, 0.57
Total power (ms²)	477.72 ± 407.79	586.97 ± 1,042.39	-0.14	-0.77, 0.48
Mean R-R (ms)	796.90 ± 132.07	807.20 ± 125.37	-0.08	-0.71, 0.54
RMSSD (ms)	17.08 ± 9.91	13.49 ± 7.03	0.43	-0.20, 1.1
SDNN (ms)	20.22 ± 8.44	19.75 ± 14.52	0.04	-0.58, 0.66
PNS index	-1.24 ± 0.88	-1.34 ± 0.70	0.12	-0.50, 0.74
SNS index	2.42 ± 1.90	2.86 ± 2.58	-0.20	-0.82, 0.42
Stress index	20.03 ± 7.32	23.15 ± 13.06	-0.30	-0.93, 0.32

The table shows the standardized mean difference between females and males in regards to subject characteristics and 95% confidence interval.

### Time-domain HRV indices

#### RMSSD

RMSSD did not show a consistent main effect of acute exercise (β = -0.1, 95% CI [-0.56, 0.38], pd = 67.1%, ps = 49.9%). However, the acute RMSSD response was modestly moderated by age, such that increasing age was associated with an inversion of the observed effect following exercise (β = 0.27, 95% CI [-0.15, 0.71], pd = 90.5%, ps = 80.4%). Sex also moderated the acute response with some degree of uncertainty, with males showing a greater RMSSD reduction after exercise compared to females (β = -0.61, 95% CI [-1.73, 0.5], pd = 86.5%, ps = 82.4%).

Additionally, body composition influenced RMSSD dynamics. Higher total fat mass was associated with larger post-exercise RMSSD increases (β = 0.5, 95% CI [-0.14, 1.13], pd = 94.1%, ps = 89.9%).

#### SDNN

No robust main effects of intervention phase or acute exercise were observed for SDNN. However, sex moderated the acute response, with males showing a larger SDNN decrease following exercise compared to females (β = -0.65, 95% CI [-2.15, 0.87], pd = 80.8%, ps = 77%).

#### Mean RR

Mean RR exhibited acute effects to exercise onset. Acute exercise was associated with a decrease in mean RR (β = -0.58, 95% CI [-1.07, -0.1], pd = 98.9%, ps = 97.3%). Age strongly moderated the chronic intervention effect, such that increasing age was associated with a larger post-intervention effect in mean RR at baseline (β = -0.45, 95% CI [-0.93, 0], pd = 97.3%, ps = 93.2%). In contrast, total fat mass moderated the acute response, with higher fat mass associated with a larger post-exercise effect in mean RR (β = 0.53, 95% CI [-0.16, 1.18], pd = 94.1%, ps = 90%).

### Frequency-domain HRV indices

#### High frequency

The intervention did not show clear overall effects on HF power. However, more aged individuals displayed a tendency to have a lower overall HF power (β = -0.11, 95% CI [-0.5, 0.27], pd = 72.3%, ps = 53.5%). After the intervention, HF increased more in response to acute exercise (β = 0.37, 95% CI [-0.38, 1.1], pd = 84.3%, ps = 76.9%).

#### Low frequency

LF power differed by sex, with males showing higher values overall (β = 0.4, 95% CI [-0.69, 1.5], pd = 76.7%, ps = 70.6%) and a larger acute decrease following exercise (β = -0.8, 95% CI [-2.13, 0.53], pd = 88.3%, ps = 85.2%). Additional moderate interactions indicated that age and body composition influenced both acute and chronic LF responses. No intervention effects were found.

#### Very low frequency

VLF power showed a larger acute exercise-associated increase after the intervention (β = 0.94, 95% CI [-0.04, 1.94], pd = 96.8%, ps = 95.1%). This interaction was potentially moderated by sex, with males exhibiting a smaller post-intervention acute VLF increase compared to females (β = -1.06, 95% CI [-3.1, 0.98], pd = 84.6%, ps = 82%).

### Autonomic composite indices

#### Parasympathetic nervous system index

Acute exercise was associated with a decrease in the PNS index (β = −0.55, 95% CI [-0.87, -0.22], pd = 99.9%, ps = 99.6%). This acute reduction was weakly moderated by age, such that increasing age was associated with a smaller post-exercise PNS decrease (β = 0.13, 95% CI [-0.15, 0.42], pd = 81%, ps = 57.1%).

#### Sympathetic nervous system index

A strong chronic intervention effect was observed for the SNS index, with SNS values being higher in the post-intervention phase compared to pre-intervention (β = 1.79, 95% CI [1.24, 2.33], pd = 100%, ps = 100%). After the intervention, participants showed a smaller exercise-induced SNS increase (β = -1.77, 95% CI [-2.52, -1.03], pd = 100%, ps = 100%).

This acute response was further moderated by body composition. Higher total fat mass was associated with larger acute SNS responses across phases (β = 0.92, 95% CI [-0.11, 1.94], pd = 96%, ps = 94.1%), whereas higher total muscle mass was associated with attenuated acute SNS responses (β = -0.31, 95% CI [-1.16, 0.55], pd = 76.2%, ps = 68.4%).

A strong three-way interaction indicates that the acute HRV response (rest-to-recovery change) differed between baseline and post-intervention, and total fat mass was observed to interact with the intervention effect (β = -0.6, 95% CI [-1.29, 0.1], pd = 95.5%, ps = 92.3%), indicating that those individuals with higher fat mass could have potentially exhibited a larger acute PNS decrease after the intervention when compared with before the intervention.

#### Stress index

No chronic, acute, or moderation effects were found.

**Figure 2:**
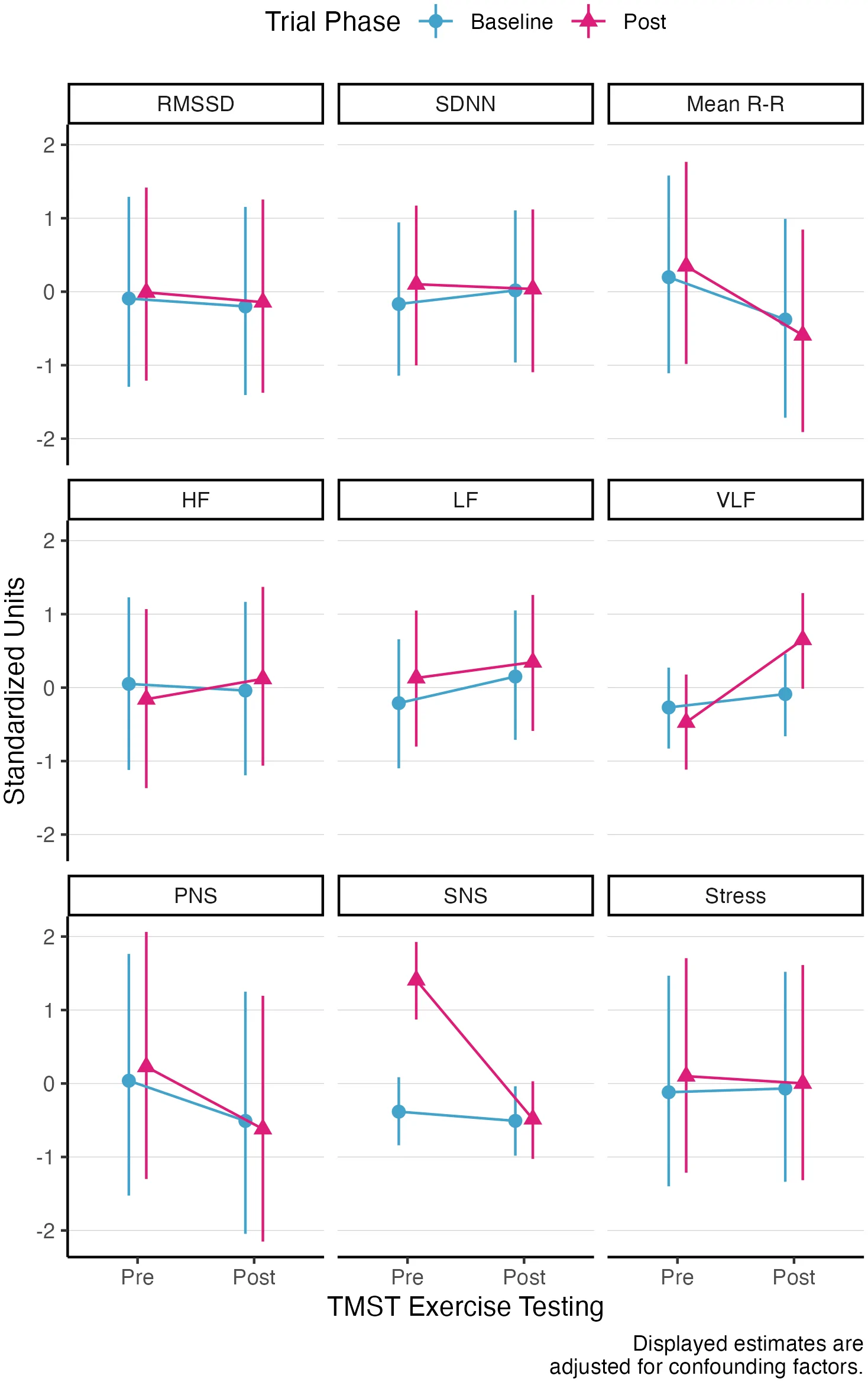
Model-predicted pre–post changes in HRV and autonomic indices during the TMST exercise test. Baseline and post-intervention measurements are superimposed to facilitate visual comparison of exercise responses across trial phases. Values are expressed in standardized units; points indicate adjusted marginal means and error bars denote 95% confidence intervals, accounting for potential confounders.

Main results are summarized in **Figure 2**.

The complete model estimates can be observed in the **Supplementary Table 1** of the **Supplementary Material**.

## Discussion

This study examined overall and exercise-induced autonomic responses following a low-dose 24-week MBE intervention in older adults living at high southern latitudes, using wearable-derived HRV measures collected under standardized laboratory conditions. Rather than observing uniform changes across HRV indices, the primary finding was a pattern of reorganization of acute autonomic responses, accompanied by substantial interindividual variability in some parameters. Across time-domain, frequency-domain, and composite autonomic indices, the intervention was associated with changes in overall HRV parameters and in the way participants responded to an acute exercise bout. These findings suggest that the physiological impact of MBE in this context is better characterized as a reorganization of autonomic responsiveness across time scales, shaped by individual characteristics, than a homogeneous effect.

The distinction between acute and chronic autonomic responses is central to interpreting these findings. Acute exercise typically induces a transient withdrawal of parasympathetic activity and a concomitant increase in sympathetic drive, followed by recovery dynamics that reflect autonomic flexibility (Furlan et al., 1993; Castillo-Aguilar et al., 2025b). In this study, the most consistent effects of the intervention emerged not as stable shifts in overall HRV, but as modifications in the magnitude and direction of acute exercise-induced responses after the intervention. Such phase-dependent modulation of acute responses has been increasingly recognized as a relevant indicator of autonomic regulation, particularly in older adults, due to their characteristic loss of resilience and adaptability (Cosarderelioglu et al., 2025).

When examined at the level of specific autonomic domains, the observed patterns further support the notion of context-dependent modulation. The increase in composite sympathetic indices observed after the intervention should not be interpreted as a maladaptive response per se, as sympathetic activity is a necessary component of physiological readiness and exercise performance, particularly in older adults (Mabe-Castro et al., 2024a; Tekin et al., 2025). Previous work has shown that exercise-based interventions may increase sympathetic responsiveness or tone as an adaptive change and be accompanied by preserved or adaptive parasympathetic modulation (Furlan et al., 1993; Amekran and El hangouche, 2024). This interpretation benefits from the observation of absolute values before and after the intervention. Given the age-associated β-adrenergic signaling and chronotropic response attenuation, older adults may show lower basal sympathetic indices and slower heart rate; in this context, a moderate increase after the intervention can reflect a better autonomic reserve instead of overactivation. In this study, the sympathetic indices were characterized by a higher level after the intervention, along with an attenuated response to the physical exercise bout, a pattern that is consistent with a recalibrated autonomic response and the absence of sympathetic overactivation.

Time-domain HRV measures further illustrated this interpretation. RMSSD and mean RR showed limited evidence of a uniform effect of the intervention; instead, their acute responses were shaped by participant characteristics, including age and body composition. Frequency-domain indices exhibited a similar behavior. Changes in VLF power and phase-dependent modulation of HF responses suggest effects on postural, slower regulatory processes and cardiorespiratory coupling, domains that are known to be influenced by both exercise exposure and background factors (Thomas et al., 2019; Amekran and El hangouche, 2024; Cairo et al., 2024; Ma et al., 2024; Andrade et al., 2025). These results contrast with previous findings that mind-body exercise can improve resting HRV (Zou et al., 2018), which could be primarily due to the low intervention dose (once per week), or population differences (e.g., residing in high-southern latitudes).

Older adults living in these high southern latitudes are exposed to substantial seasonal fluctuations in daylight availability, cold temperatures, and weather conditions that may influence autonomic regulation through circadian, behavioral, and thermoregulatory mechanisms. Such environmental stressors have been associated with alterations in cardiovascular autonomic control and may contribute to the large interindividual variability observed in this study (Mabe-Castro et al., 2024b; Castillo-Aguilar et al., 2025a; Klos et al., 2025). Although environmental variables were not directly measured, the observed responses should be interpreted within this broader environmental context.

Indices reflecting sympathetic activity also showed distinct temporal dynamics. The model indicated a relatively strong chronic effect on the SNS index, which corresponds to a sustained upward shift in the modeled SNS-related HRV component across the intervention period. In contrast, acute responses were phase dependent, with transient modulation around this elevated baseline. Interpreted clinically, this pattern is consistent with a persistent adjustment in sympathetic tone or regulatory balance, while short-term fluctuations remain sensitive to contextual factors such as recovery state and individual characteristics (Castillo-Aguilar et al., 2021). This distinction between a stable chronic shift and phase-specific acute responses aligns with the broader pattern observed across HRV domains in the model (Alyahya et al., 2024).

An aspect to note in our findings is the consistent influence of individual characteristics on the effect of the intervention on both overall autonomic profiles and exercise-induced HRV responses following MBE. Age, sex, and body composition emerged as key modifiers across autonomic domains, supporting that autonomic adaptation to exercise-based interventions in older adults is inherently heterogeneous and markedly affected by lifestyle. Aging is accompanied by increased interindividual variability in autonomic regulation, reflecting differences in physiological reserve, cardiometabolic status, and cumulative exposure to environmental and daily life stressors (Alyahya et al., 2024; El-Malahi et al., 2024; Yelisyeyeva et al., 2025).

Age-related moderation of intervention effects is consistent with established declines in parasympathetic flexibility, baroreflex sensitivity, and recovery capacity with advancing age (de Matos et al., 2025). Body composition further shaped autonomic responses, with total fat mass moderating chronic, acute, and chronic-acute interactions, in line with evidence linking adiposity to heightened sympathetic drive and reduced autonomic adaptability (Cvijetic et al., 2023; Mabe-Castro et al., 2024a). In contrast, total muscle mass showed opposing associations in some models, supporting literature connecting greater preserved lean mass with improved autonomic responsiveness and cardiovascular regulation (Mabe-Castro et al., 2024a). Sex-related moderation was also observed, consistent with known sex differences in autonomic control and exercise responses across the lifespan (Koenig and Thayer, 2016). These findings suggest that the autonomic impact of MBE on older adults from high-southern latitudes could be influenced by individual characteristics, highlighting the relevance of considering interindividual variability when interpreting intervention effects and selecting a target population.

Finally, our findings support the feasibility of using wearable-derived HRV to characterize general and exercise-induced regulation following an MBE intervention in older adults living under environmentally challenging conditions. Although our pilot intervention did not elicit uniform shifts in resting HRV indices, the consistent modulation of acute responses and the influence of individual characteristics indicate that wearable-based assessment are sensitive to meaningful changes in autonomic regulation that may not be captured by routine resting measures alone. From a design perspective, this highlights the importance of incorporating physiological challenges and accounting for participant heterogeneity when evaluating interventions targeting autonomic health in later life. Several limitations should be acknowledged in this study, including the absence of a control group, the potential confounding effects of comorbidities, drugs or physical activity level, the modest sample size, and the laboratory-based assessment setup instead of a more ecological, real-life wearable-based follow-up. This design could constrain causal inference and generalizability. Nonetheless, the use of multivariate Bayesian modeling and the convergence of effects across autonomic domains strengthen confidence in the observed patterns. Future studies should aim to replicate these findings in controlled daily life designs, explore long-term adaptations, and integrate behavioral, environmental, and physiological data to better understand how MBE may support or even improve autonomic regulation and adaptive capacity in aging populations living under extreme environments. Moreover, these MBE interventions may be of special interest to those exposed to extreme environments in a labor or working context.

## Conclusions

In this study, a low-dose MBE intervention in older adults living at high southern latitudes was associated with changes in cardiac autonomic regulation, especially when comparing pre to post-acute autonomic reactivity to exercise testing. This pattern may reflect improved autonomic flexibility or stress reactivity, suggesting that the intervention primarily influenced the dynamic capacity of the autonomic nervous system to respond to physiological demands. Wearable-based HRV monitoring enabled practical and feasible data collection in this geographically remote setting, allowing these response patterns to be captured under standardized conditions. Age, sex, and body composition also emerged as important modulators of the intervention response, highlighting the relevance of individual physiological heterogeneity when evaluating autonomic adaptations in older populations.

## Data Availability

The raw data supporting the conclusions of this article will be made available by the authors, without undue reservation.
