# Supplementary material for: Wearable-derived heart rate variability responses to a mind-body exercise intervention in older people from high southern latitudes (Chilean Patagonia, ~53°S)
Source: Front Physiol. 2026 Aug 28;17:1794752. doi: 10.3389/fphys.2026.1794752 (PMC13561979; doi:10.3389/fphys.2026.1794752)
Supplement: Supplementary file 1 [file DataSheet1.pdf]

Supplementary Material

Title: Wearable-derived Heart Rate Variability responses to a Mind-Body Exercise Intervention in Older People from High Southern Latitudes (Chilean Patagonia, ~53°S)

Authors: Diego Mabe-Castro[1], Ruby Méndez-Muñoz[2], Pablo Sanhueza[2], Matías Castillo-Aguilar[2], Ginés Viscor[3], Antonio Zamuner[4,5], Cristian Núñez-Espinosa[2*]

[1]Centro Centro Asistencial Docente e Investigación, Universidad de Magallanes (UMAG), Punta Arenas, Chile

[2]Escuela de Medicina, Universidad de Magallanes (UMAG), Punta Arenas, Chile

[3]Secció de Fisiologia, Departament de Biologia Cel·lular, Fisiologia i Immunologia, Facultat de Biologia, Universitat de Barcelona, Barcelona, Spain

[4]Laboratorio de Investigación Clínica en Kinesiología, Departamento de Kinesiología, Universidad Católica del Maule, Talca, Chile

[5]Centro de Investigación en Neuropsicología y Neurociencias (CINPSI Neurocog), Universidad Católica del Maule, Talca Chile

***Correspondence**: Cristian Núñez-Espinosa, Escuela de Medicina, Universidad de Magallanes, Punta Arenas, Chile. Centro Asistencial de Docencia e Investigación CADI-UMAG, Chile. e-mail: cristian.nunez@umag.cl Postal address: Avenida Bulnes 01855, Box 113-D. Phone: +56 61 2201411.

Model Estimates

Table S1. Supplementary material table with the complete model estimates and diagnostics per model parameter with complete posterior distribution indices. All models are adjusted for confounding factors. $CI_{95\%}$, credible interval based on highest density interval (HDI); pd, probability of direction; ps, probability of significance; ESS, effective sample size; R-hat, potential scale reduction factor.

Parameter	Estimate	$CI_{95\%}$	pd	ps	ESS	R-hat
b_rmssd_Intercept	-0.04	[-0.49, 0.42]	0.574	0.403	8,874.5	1.000
b_sdnn_Intercept	-0.15	[-0.66, 0.36]	0.717	0.573	8,430.5	1.000
b_meanrr_Intercept	0.20	[-0.26, 0.69]	0.799	0.664	10,895.2	1.000
b_rmssd_trial_phasePost	0.09	[-0.5, 0.67]	0.615	0.482	7,770.4	1.000
b_rmssd_timePost	-0.10	[-0.56, 0.38]	0.671	0.499	6,852.4	1.000
b_rmssd_edad	-0.19	[-0.57, 0.19]	0.842	0.684	9,295.6	1.000
b_rmssd_sd_sexoMasculino	0.45	[-0.62, 1.49]	0.799	0.744	7,332.4	1.000
b_rmssd_fat_total	-0.38	[-0.98, 0.19]	0.903	0.828	7,155.5	1.001
b_rmssd_muscle_total	-0.34	[-0.9, 0.23]	0.879	0.797	4,439.0	1.001
b_rmssd_imc	0.48	[-0.13, 1.11]	0.937	0.886	7,115.2	1.001
b_rmssd_trial_phasePost:timePost	-0.04	[-0.74, 0.68]	0.551	0.434	6,789.2	1.000
b_rmssd_trial_phasePost:edad	-0.32	[-0.76, 0.13]	0.928	0.841	5,968.3	1.000
b_rmssd_trial_phasePost:sd_sexoMasculino	0.16	[-1.15, 1.49]	0.594	0.537	7,094.3	1.000
b_rmssd_trial_phasePost:fat_total	0.51	[-0.27, 1.28]	0.903	0.852	7,597.0	1.000
b_rmssd_trial_phasePost:muscle_total	-0.09	[-0.79, 0.61]	0.593	0.482	6,035.6	1.001
b_rmssd_trial_phasePost:imc	-0.64	[-1.48, 0.25]	0.929	0.892	6,509.0	1.001

b_rmssd_timePost:edad	0.27	[-0.15, 0.71]	0.905	0.804	9,205.7	1.000
b_rmssd_timePost:sd_sexoMasculino	-0.61	[-1.73, 0.5]	0.865	0.824	5,453.7	1.000
b_rmssd_timePost:fat_total	0.50	[-0.14, 1.13]	0.941	0.899	7,042.8	1.000
b_rmssd_timePost:muscle_total	0.04	[-0.56, 0.61]	0.556	0.415	5,357.3	1.000
b_rmssd_timePost:imc	-0.17	[-0.86, 0.56]	0.696	0.586	5,786.3	1.001
b_rmssd_trial_phasePost:timePost:edad	0.01	[-0.57, 0.58]	0.517	0.371	8,450.3	1.000
b_rmssd_trial_phasePost:timePost:sd_sexoMasculino	0.56	[-0.94, 2.01]	0.781	0.742	5,625.1	1.000
b_rmssd_trial_phasePost:timePost:fat_total	-0.32	[-1.3, 0.65]	0.750	0.680	7,095.0	1.000
b_rmssd_trial_phasePost:timePost:muscle_total	-0.04	[-0.84, 0.76]	0.554	0.451	5,798.1	1.000
b_rmssd_trial_phasePost:timePost:imc	0.11	[-0.95, 1.18]	0.592	0.516	6,045.5	1.001
b_sdnn_trial_phasePost	0.24	[-0.48, 1.02]	0.738	0.644	6,201.3	1.000
b_sdnn_timePost	0.20	[-0.44, 0.83]	0.737	0.626	7,800.0	1.000
b_sdnn_edad	-0.24	[-0.69, 0.22]	0.846	0.726	6,598.5	1.000
b_sdnn_sd_sexoMasculino	0.24	[-1.02, 1.47]	0.652	0.592	5,658.2	1.001
b_sdnn_fat_total	0.11	[-0.57, 0.78]	0.626	0.509	7,222.9	1.001
b_sdnn_muscle_total	-0.14	[-0.77, 0.49]	0.669	0.545	7,039.4	1.001
b_sdnn_imc	-0.19	[-0.94, 0.58]	0.687	0.590	5,594.2	1.001
b_sdnn_trial_phasePost:timePost	-0.26	[-1.2, 0.7]	0.715	0.641	7,363.9	1.000
b_sdnn_trial_phasePost:edad	-0.37	[-0.97, 0.22]	0.889	0.810	4,976.3	1.000
b_sdnn_trial_phasePost:sd_sexoMasculino	-0.14	[-1.81, 1.41]	0.565	0.517	4,937.2	1.000

b_sdnn_trial_phasePost:fat_total	-0.32	[-1.28, 0.7]	0.739	0.674	7,901.3	1.001
b_sdnn_trial_phasePost:muscle_total	0.06	[-0.8, 0.89]	0.554	0.462	6,565.5	1.001
b_sdnn_trial_phasePost:imc	-0.08	[-1.16, 1.04]	0.550	0.475	6,020.1	1.001
b_sdnn_timePost:edad	0.13	[-0.44, 0.73]	0.679	0.539	9,741.9	1.000
b_sdnn_timePost:sd_sexoMasculino	-0.65	[-2.15, 0.87]	0.808	0.770	5,982.8	1.000
b_sdnn_timePost:fat_total	0.26	[-0.6, 1.13]	0.732	0.650	7,636.4	1.000
b_sdnn_timePost:muscle_total	0.20	[-0.58, 0.99]	0.702	0.607	5,889.4	1.000
b_sdnn_timePost:imc	-0.10	[-1.07, 0.86]	0.583	0.498	6,306.5	1.001
b_sdnn_trial_phasePost:timePost:edad	0.02	[-0.75, 0.79]	0.522	0.414	9,068.5	1.000
b_sdnn_trial_phasePost:timePost:sd_sexoMasculino	1.04	[-0.95, 2.98]	0.851	0.828	6,140.7	1.000
b_sdnn_trial_phasePost:timePost:fat_total	0.15	[-1.19, 1.47]	0.583	0.523	7,826.3	1.000
b_sdnn_trial_phasePost:timePost:muscle_total	-0.39	[-1.45, 0.71]	0.766	0.707	6,276.6	1.000
b_sdnn_trial_phasePost:timePost:imc	-0.05	[-1.49, 1.38]	0.525	0.470	6,715.7	1.001
b_meanrr_trial_phasePost	0.16	[-0.46, 0.82]	0.684	0.567	9,539.7	1.000
b_meanrr_timePost	-0.58	[-1.07, -0.1]	0.989	0.973	8,806.1	1.000
b_meanrr_edad	0.15	[-0.25, 0.56]	0.768	0.597	11,416.8	1.000
b_meanrr_sd_sexoMasculino	0.76	[-0.37, 1.88]	0.906	0.875	9,385.9	1.000
b_meanrr_fat_total	0.11	[-0.51, 0.74]	0.635	0.514	8,328.3	1.000
b_meanrr_muscle_total	-0.13	[-0.7, 0.45]	0.663	0.535	9,532.2	1.001
b_meanrr_imc	-0.04	[-0.73, 0.64]	0.549	0.435	7,861.2	1.000

b_meanrr_trial_phasePost:timePost	-0.38	[-1.13, 0.37]	0.841	0.768	9,528.2	1.000
b_meanrr_trial_phasePost:edad	-0.45	[-0.93, 0]	0.973	0.932	11,680.2	1.000
b_meanrr_trial_phasePost:sd_sexoMasculino	-0.10	[-1.58, 1.43]	0.554	0.502	8,850.8	1.000
b_meanrr_trial_phasePost:fat_total	-0.54	[-1.36, 0.35]	0.896	0.847	8,475.8	1.000
b_meanrr_trial_phasePost:muscle_total	-0.46	[-1.24, 0.3]	0.881	0.825	8,204.2	1.001
b_meanrr_trial_phasePost:imc	0.46	[-0.47, 1.41]	0.832	0.775	7,463.7	1.000
b_meanrr_timePost:edad	0.05	[-0.4, 0.48]	0.586	0.404	11,124.8	1.000
b_meanrr_timePost:sd_sexoMasculino	-0.23	[-1.42, 0.97]	0.645	0.583	7,489.7	1.000
b_meanrr_timePost:fat_total	0.53	[-0.16, 1.18]	0.941	0.900	9,432.6	1.000
b_meanrr_timePost:muscle_total	-0.06	[-0.66, 0.57]	0.578	0.448	7,572.0	1.001
b_meanrr_timePost:imc	-0.43	[-1.19, 0.31]	0.873	0.811	8,139.3	1.000
b_meanrr_trial_phasePost:timePost:edad	0.26	[-0.34, 0.84]	0.811	0.706	11,152.8	1.000
b_meanrr_trial_phasePost:timePost:sd_sexoMasculino	-0.29	[-1.89, 1.26]	0.641	0.593	8,135.1	1.000
b_meanrr_trial_phasePost:timePost:fat_total	-0.29	[-1.32, 0.76]	0.713	0.646	9,255.8	1.000
b_meanrr_trial_phasePost:timePost:muscle_total	0.40	[-0.45, 1.24]	0.823	0.757	8,317.2	1.001
b_meanrr_trial_phasePost:timePost:imc	0.09	[-1.05, 1.24]	0.563	0.493	8,335.4	1.000
b_hf_Intercept	0.07	[-0.38, 0.53]	0.611	0.441	9,502.5	1.000
b_lf_Intercept	-0.21	[-0.65, 0.24]	0.820	0.682	9,114.0	1.000
b_vlf_Intercept	-0.27	[-0.76, 0.2]	0.868	0.760	10,187.4	1.000

b_hf_trial_phasePost	-0.21	[-0.83, 0.4]	0.748	0.637	8,883.2	1.000
b_hf_timePost	-0.09	[-0.58, 0.4]	0.647	0.486	8,992.2	1.000
b_hf_edad	-0.11	[-0.5, 0.27]	0.723	0.535	10,151.4	1.000
b_hf_sd_sexoMasculino	-0.05	[-1.13, 1.06]	0.541	0.470	7,401.9	1.000
b_hf_fat_total	0.00	[-0.61, 0.61]	0.504	0.376	7,949.5	1.000
b_hf_muscle_total	-0.04	[-0.59, 0.54]	0.554	0.414	7,345.9	1.000
b_hf_imc	0.03	[-0.64, 0.69]	0.536	0.417	7,362.8	1.000
b_hf_trial_phasePost:timePost	0.37	[-0.38, 1.1]	0.843	0.769	8,774.5	1.000
b_hf_trial_phasePost:edad	-0.41	[-0.88, 0.04]	0.962	0.909	9,863.3	1.000
b_hf_trial_phasePost:sd_sexoMasculino	0.90	[-0.55, 2.27]	0.893	0.866	7,352.6	1.000
b_hf_trial_phasePost:fat_total	0.45	[-0.37, 1.27]	0.861	0.802	8,485.2	1.000
b_hf_trial_phasePost:muscle_total	-0.62	[-1.36, 0.12]	0.949	0.916	7,179.5	1.000
b_hf_trial_phasePost:imc	-0.43	[-1.36, 0.46]	0.823	0.760	7,468.4	1.000
b_hf_timePost:edad	0.30	[-0.14, 0.72]	0.916	0.828	9,667.4	1.000
b_hf_timePost:sd_sexoMasculino	-0.32	[-1.45, 0.91]	0.706	0.646	6,483.6	1.000
b_hf_timePost:fat_total	0.25	[-0.4, 0.92]	0.777	0.676	7,750.7	1.000
b_hf_timePost:muscle_total	0.04	[-0.58, 0.65]	0.556	0.425	7,054.3	1.000
b_hf_timePost:imc	-0.06	[-0.81, 0.67]	0.571	0.464	6,830.8	1.000
b_hf_trial_phasePost:timePost:edad	-0.13	[-0.72, 0.46]	0.671	0.538	9,902.6	1.000
b_hf_trial_phasePost:timePost:sd_sexoMasculino	-0.15	[-1.74, 1.36]	0.576	0.524	6,574.8	1.000

b_hf_trial_phasePost:timePost:fat_total	-0.25	[-1.24, 0.8]	0.690	0.617	8,145.3	1.000
b_hf_trial_phasePost:timePost:muscle_total	0.21	[-0.64, 1.04]	0.686	0.598	7,033.4	1.000
b_hf_trial_phasePost:timePost:imc	0.01	[-1.09, 1.15]	0.508	0.437	7,291.7	1.000
b_lf_trial_phasePost	0.34	[-0.32, 0.98]	0.847	0.765	8,246.2	1.000
b_lf_timePost	0.36	[-0.19, 0.93]	0.903	0.828	7,984.9	1.000
b_lf_edad	-0.10	[-0.49, 0.3]	0.688	0.495	9,553.7	1.000
b_lf_sd_sexoMasculino	0.40	[-0.69, 1.5]	0.767	0.706	6,239.6	1.000
b_lf_fat_total	0.28	[-0.33, 0.89]	0.824	0.727	6,932.8	1.000
b_lf_muscle_total	-0.20	[-0.76, 0.35]	0.766	0.646	6,192.5	1.000
b_lf_imc	-0.39	[-1.06, 0.27]	0.877	0.807	6,126.5	1.000
b_lf_trial_phasePost:timePost	-0.15	[-0.97, 0.69]	0.644	0.549	7,670.6	1.000
b_lf_trial_phasePost:edad	-0.42	[-0.94, 0.1]	0.949	0.890	8,643.8	1.000
b_lf_trial_phasePost:sd_sexoMasculino	-0.63	[-2.12, 0.79]	0.803	0.763	6,473.0	1.000
b_lf_trial_phasePost:fat_total	-0.62	[-1.49, 0.28]	0.914	0.874	7,011.0	1.000
b_lf_trial_phasePost:muscle_total	0.22	[-0.51, 1]	0.718	0.629	5,960.9	1.000
b_lf_trial_phasePost:imc	0.35	[-0.65, 1.31]	0.764	0.699	6,222.5	1.000
b_lf_timePost:edad	-0.17	[-0.65, 0.35]	0.747	0.605	9,240.4	1.000
b_lf_timePost:sd_sexoMasculino	-0.80	[-2.13, 0.53]	0.883	0.852	5,616.7	1.000
b_lf_timePost:fat_total	-0.05	[-0.8, 0.69]	0.548	0.444	6,842.8	1.000
b_lf_timePost:muscle_total	0.20	[-0.49, 0.88]	0.719	0.614	5,845.9	1.000

b_lf_timePost:imc	0.18	[-0.65, 1.01]	0.673	0.581	5,765.6	1.000
b_lf_trial_phasePost:timePost:edad	0.30	[-0.37, 0.97]	0.812	0.725	9,405.2	1.000
b_lf_trial_phasePost:timePost:sd_sexoMasculino	0.72	[-1.1, 2.41]	0.797	0.762	5,840.7	1.000
b_lf_trial_phasePost:timePost:fat_total	0.05	[-1.08, 1.2]	0.536	0.468	7,284.6	1.000
b_lf_trial_phasePost:timePost:muscle_total	-0.63	[-1.58, 0.33]	0.904	0.863	5,745.6	1.000
b_lf_trial_phasePost:timePost:imc	0.05	[-1.21, 1.31]	0.532	0.466	6,144.5	1.000
b_vlf_trial_phasePost	-0.20	[-0.92, 0.53]	0.708	0.605	9,379.9	1.000
b_vlf_timePost	0.19	[-0.49, 0.83]	0.713	0.605	8,476.0	1.000
b_vlf_edad	0.02	[-0.4, 0.46]	0.547	0.365	10,153.4	1.000
b_vlf_sd_sexoMasculino	1.07	[-0.08, 2.21]	0.966	0.951	6,789.9	1.000
b_vlf_fat_total	0.27	[-0.39, 0.9]	0.795	0.701	7,981.2	1.000
b_vlf_muscle_total	-0.45	[-1.03, 0.16]	0.932	0.881	6,793.4	1.000
b_vlf_imc	-0.30	[-1.03, 0.43]	0.798	0.713	6,907.7	1.000
b_vlf_trial_phasePost:timePost	0.94	[-0.04, 1.94]	0.968	0.951	8,439.6	1.000
b_vlf_trial_phasePost:edad	-0.44	[-1.05, 0.12]	0.932	0.875	10,506.4	1.000
b_vlf_trial_phasePost:sd_sexoMasculino	-0.20	[-1.73, 1.32]	0.602	0.552	7,299.2	1.000
b_vlf_trial_phasePost:fat_total	0.00	[-1.02, 0.97]	0.496	0.417	8,297.8	1.000
b_vlf_trial_phasePost:muscle_total	-0.17	[-1.01, 0.64]	0.658	0.565	6,674.6	1.000
b_vlf_trial_phasePost:imc	0.19	[-0.89, 1.29]	0.637	0.567	7,156.6	1.000
b_vlf_timePost:edad	-0.01	[-0.63, 0.57]	0.523	0.391	9,657.8	1.000

b_vlf_timePost:sd_sexoMasculino	-0.50	[-2.07, 1.02]	0.740	0.697	5,847.2	1.000
b_vlf_timePost:fat_total	0.12	[-0.77, 1.01]	0.600	0.513	7,423.3	1.000
b_vlf_timePost:muscle_total	0.30	[-0.56, 1.07]	0.768	0.688	5,879.9	1.000
b_vlf_timePost:imc	0.09	[-0.88, 1.1]	0.576	0.499	6,166.1	1.000
b_vlf_trial_phasePost:timePost:edad	-0.05	[-0.84, 0.76]	0.550	0.449	9,883.3	1.000
b_vlf_trial_phasePost:timePost:sd_sexoMasculino	-1.06	[-3.1, 0.98]	0.846	0.820	6,440.8	1.000
b_vlf_trial_phasePost:timePost:fat_total	-0.32	[-1.69, 1.01]	0.682	0.627	7,898.8	1.000
b_vlf_trial_phasePost:timePost:muscle_total	0.41	[-0.72, 1.53]	0.763	0.705	6,100.9	1.000
b_vlf_trial_phasePost:timePost:imc	-0.10	[-1.59, 1.38]	0.555	0.500	6,637.8	1.000
b_pns_Intercept	0.10	[-0.36, 0.58]	0.659	0.499	10,093.3	1.000
b_sns_Intercept	-0.38	[-0.74, -0.02]	0.980	0.938	16,560.5	1.000
b_stress_Intercept	-0.06	[-0.51, 0.38]	0.609	0.433	12,043.7	1.000
b_pns_trial_phasePost	0.20	[-0.31, 0.71]	0.776	0.644	11,750.7	1.000
b_pns_timePost	-0.55	[-0.87, -0.22]	0.999	0.996	13,849.3	1.000
b_pns_edad	0.00	[-0.39, 0.38]	0.510	0.308	11,616.2	1.000
b_pns_sd_sexoMasculino	0.88	[-0.22, 1.96]	0.945	0.921	9,979.8	1.000
b_pns_fat_total	-0.28	[-0.85, 0.32]	0.828	0.729	11,754.1	1.000
b_pns_muscle_total	-0.45	[-1, 0.09]	0.950	0.900	10,464.1	1.000
b_pns_imc	0.43	[-0.15, 1.02]	0.929	0.872	12,026.3	1.000
b_pns_trial_phasePost:timePost	-0.30	[-0.81, 0.21]	0.883	0.788	14,558.3	1.000

b_pns_trial_phasePost:edad	-0.33	[-0.67, 0]	0.975	0.915	15,668.0	1.000
b_pns_trial_phasePost:sd_sexoMasculino	-0.08	[-1.36, 1.22]	0.548	0.487	10,631.5	1.000
b_pns_trial_phasePost:fat_total	0.04	[-0.59, 0.69]	0.552	0.430	13,061.2	1.000
b_pns_trial_phasePost:muscle_total	-0.20	[-0.85, 0.43]	0.730	0.619	10,606.1	1.000
b_pns_trial_phasePost:imc	-0.07	[-0.79, 0.66]	0.573	0.464	11,592.2	1.000
b_pns_timePost:edad	0.13	[-0.15, 0.42]	0.810	0.571	21,425.5	1.000
b_pns_timePost:sd_sexoMasculino	-0.41	[-1.22, 0.41]	0.838	0.772	11,215.8	1.000
b_pns_timePost:fat_total	0.65	[0.21, 1.09]	0.997	0.991	14,844.6	1.000
b_pns_timePost:muscle_total	-0.03	[-0.44, 0.38]	0.554	0.363	11,844.2	1.000
b_pns_timePost:imc	-0.44	[-0.95, 0.05]	0.957	0.909	12,280.8	1.000
b_pns_trial_phasePost:timePost:edad	0.16	[-0.23, 0.54]	0.798	0.626	20,110.2	1.000
b_pns_trial_phasePost:timePost:sd_sexoMasculino	0.23	[-0.86, 1.37]	0.658	0.591	12,072.7	1.000
b_pns_trial_phasePost:timePost:fat_total	-0.60	[-1.29, 0.1]	0.955	0.923	15,004.8	1.000
b_pns_trial_phasePost:timePost:muscle_total	0.21	[-0.38, 0.8]	0.759	0.644	12,845.5	1.000
b_pns_trial_phasePost:timePost:imc	0.52	[-0.23, 1.31]	0.911	0.865	12,866.4	1.000
b_sns_trial_phasePost	1.79	[1.24, 2.33]	1.000	1.000	14,507.7	1.000
b_sns_timePost	-0.13	[-0.62, 0.35]	0.697	0.543	15,089.3	1.000
b_sns_edad	0.14	[-0.18, 0.45]	0.809	0.603	19,160.0	1.000
b_sns_sd_sexoMasculino	0.40	[-0.5, 1.28]	0.814	0.749	12,171.4	1.000
b_sns_fat_total	0.26	[-0.23, 0.74]	0.853	0.741	13,230.6	1.000

b_sns_muscle_total	-0.21	[-0.68, 0.22]	0.826	0.691	12,686.5	1.000
b_sns_imc	-0.08	[-0.64, 0.45]	0.620	0.476	11,623.9	1.000
b_sns_trial_phasePost:timePost	-1.77	[-2.52, -1.03]	1.000	1.000	14,221.1	1.000
b_sns_trial_phasePost:edad	-0.21	[-0.63, 0.21]	0.834	0.696	19,612.3	1.000
b_sns_trial_phasePost:sd_sexoMasculino	-0.41	[-1.59, 0.8]	0.752	0.696	11,690.6	1.000
b_sns_trial_phasePost:fat_total	-0.91	[-1.68, -0.15]	0.990	0.980	13,880.8	1.000
b_sns_trial_phasePost:muscle_total	0.38	[-0.24, 1.02]	0.881	0.809	12,627.3	1.000
b_sns_trial_phasePost:imc	0.46	[-0.39, 1.29]	0.865	0.806	12,548.3	1.000
b_sns_timePost:edad	-0.15	[-0.6, 0.28]	0.756	0.592	19,782.7	1.000
b_sns_timePost:sd_sexoMasculino	-0.40	[-1.64, 0.77]	0.743	0.690	11,728.8	1.000
b_sns_timePost:fat_total	-0.29	[-0.94, 0.38]	0.804	0.712	13,484.0	1.000
b_sns_timePost:muscle_total	0.21	[-0.41, 0.82]	0.748	0.632	12,519.6	1.000
b_sns_timePost:imc	0.10	[-0.65, 0.84]	0.610	0.503	11,777.8	1.000
b_sns_trial_phasePost:timePost:edad	0.24	[-0.35, 0.82]	0.794	0.683	20,007.3	1.000
b_sns_trial_phasePost:timePost:sd_sexoMasculino	0.34	[-1.26, 1.97]	0.661	0.616	11,391.3	1.000
b_sns_trial_phasePost:timePost:fat_total	0.92	[-0.11, 1.94]	0.960	0.941	13,790.8	1.000
b_sns_trial_phasePost:timePost:muscle_total	-0.31	[-1.16, 0.55]	0.762	0.684	12,794.0	1.000
b_sns_trial_phasePost:timePost:imc	-0.49	[-1.59, 0.68]	0.801	0.749	12,476.8	1.000
b_stress_trial_phasePost	0.22	[-0.33, 0.76]	0.790	0.667	11,540.4	1.000
b_stress_timePost	0.05	[-0.33, 0.42]	0.602	0.390	13,859.3	1.000

b_stress_edad	0.19	[-0.19, 0.54]	0.845	0.681	14,101.4	1.000
b_stress_sd_sexoMasculino	-0.19	[-1.25, 0.84]	0.639	0.568	11,310.8	1.000
b_stress_fat_total	-0.32	[-0.89, 0.27]	0.859	0.770	11,803.0	1.000
b_stress_muscle_total	0.21	[-0.34, 0.73]	0.777	0.652	11,579.3	1.000
b_stress_imc	0.24	[-0.37, 0.84]	0.779	0.671	11,579.7	1.000
b_stress_trial_phasePost:timePost	-0.15	[-0.74, 0.42]	0.699	0.567	14,714.9	1.000
b_stress_trial_phasePost:edad	0.24	[-0.12, 0.63]	0.904	0.775	14,964.6	1.000
b_stress_trial_phasePost:sd_sexoMasculino	-0.34	[-1.68, 0.95]	0.690	0.637	10,855.7	1.000
b_stress_trial_phasePost:fat_total	0.46	[-0.23, 1.16]	0.905	0.848	13,444.4	1.000
b_stress_trial_phasePost:muscle_total	0.34	[-0.32, 1.01]	0.841	0.760	11,118.2	1.000
b_stress_trial_phasePost:imc	-0.47	[-1.23, 0.32]	0.884	0.828	11,244.2	1.000
b_stress_timePost:edad	-0.15	[-0.47, 0.18]	0.815	0.610	21,516.6	1.000
b_stress_timePost:sd_sexoMasculino	0.40	[-0.51, 1.34]	0.806	0.745	11,633.3	1.000
b_stress_timePost:fat_total	-0.21	[-0.72, 0.27]	0.805	0.675	14,903.7	1.000
b_stress_timePost:muscle_total	-0.11	[-0.58, 0.36]	0.673	0.513	12,187.6	1.000
b_stress_timePost:imc	0.13	[-0.43, 0.7]	0.677	0.540	12,479.1	1.000
b_stress_trial_phasePost:timePost:edad	-0.04	[-0.46, 0.41]	0.579	0.394	20,871.8	1.000
b_stress_trial_phasePost:timePost:sd_sexoMasculino	-0.31	[-1.59, 0.92]	0.688	0.630	11,993.1	1.000
b_stress_trial_phasePost:timePost:fat_total	-0.33	[-1.11, 0.46]	0.795	0.719	15,191.2	1.000
b_stress_trial_phasePost:timePost:muscle_total	0.09	[-0.56, 0.75]	0.609	0.490	12,775.9	1.000

b_stress_trial_phasePost:timePost:imc	0.16	[-0.71, 1.02]	0.647	0.557	12,995.5	1.000
